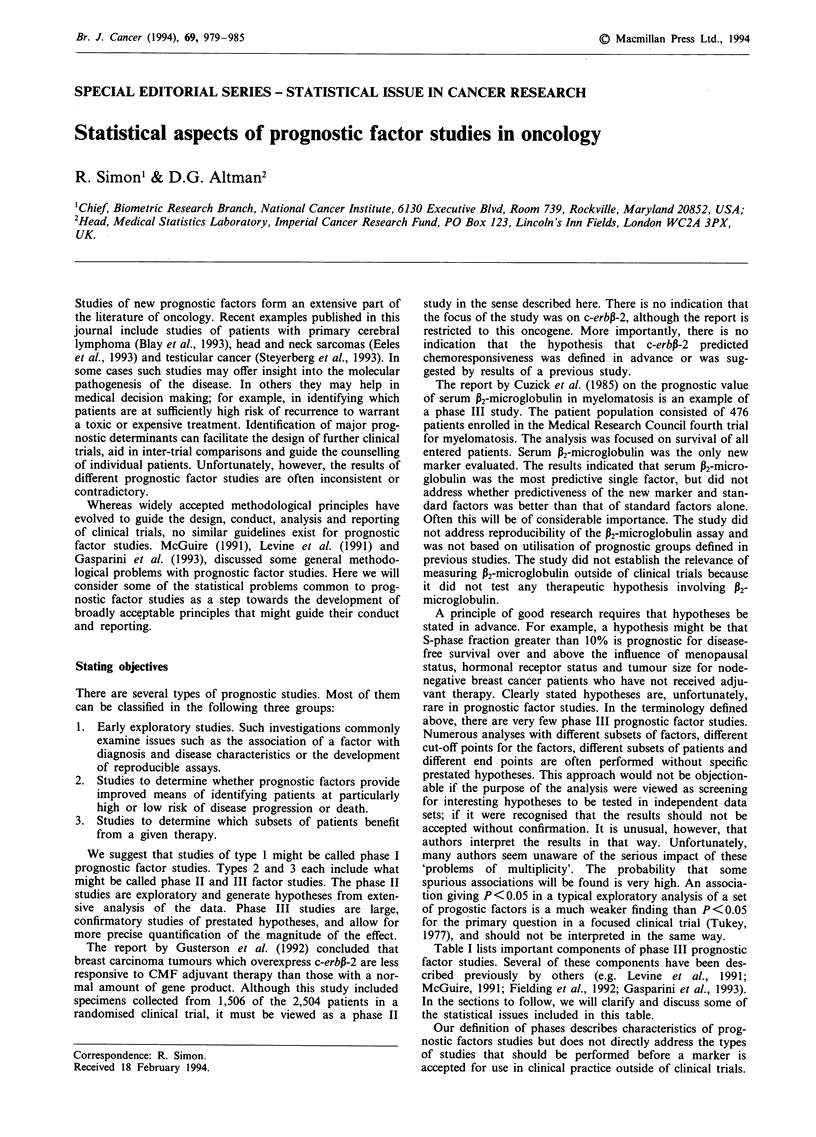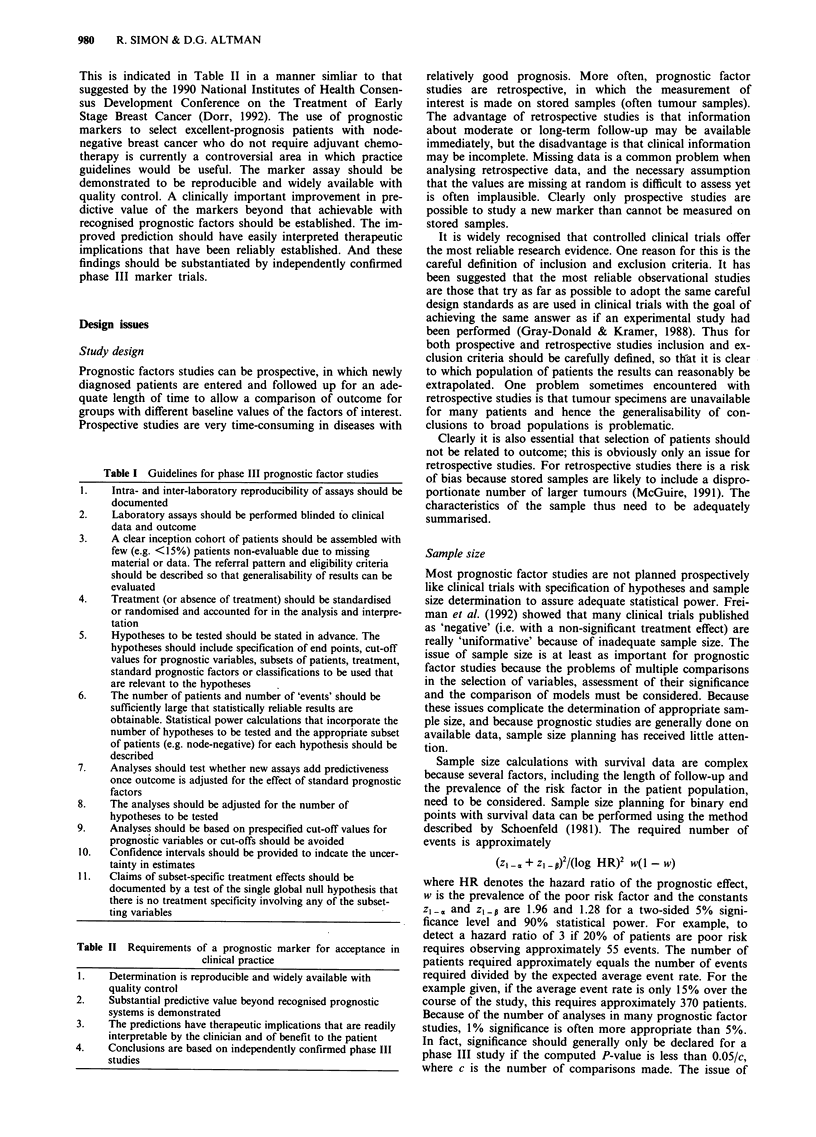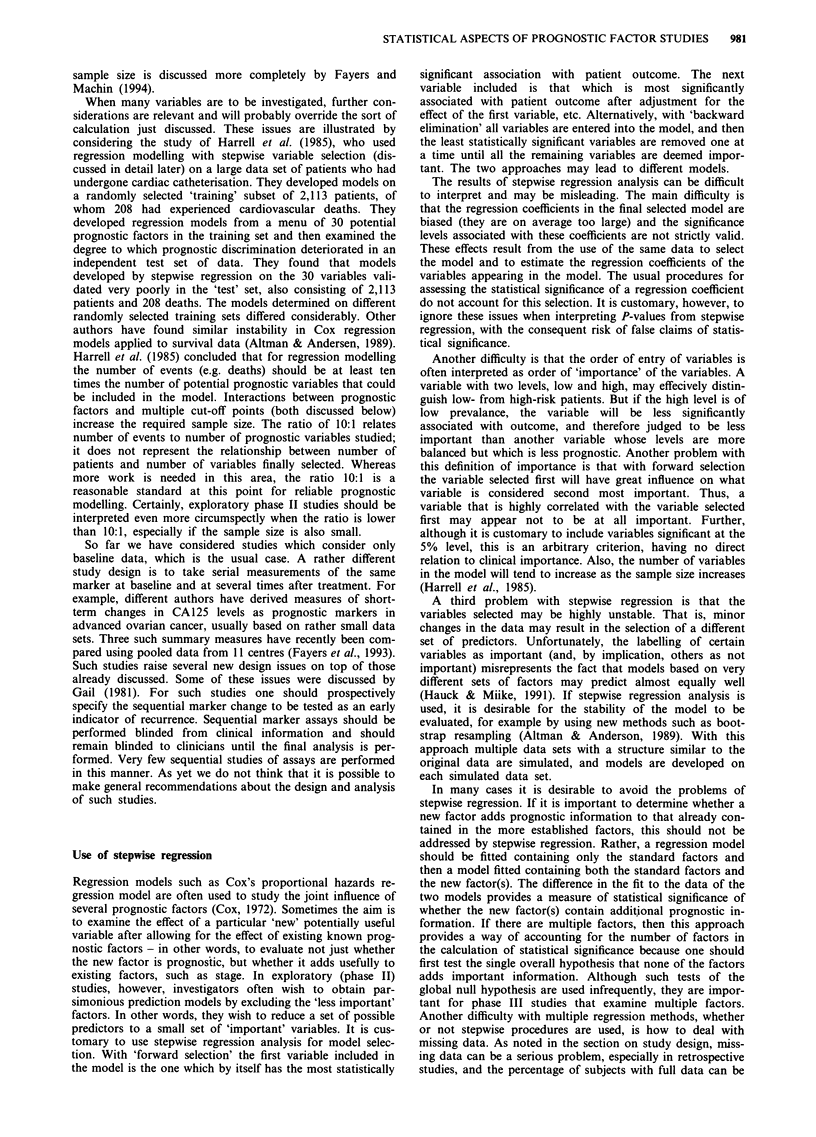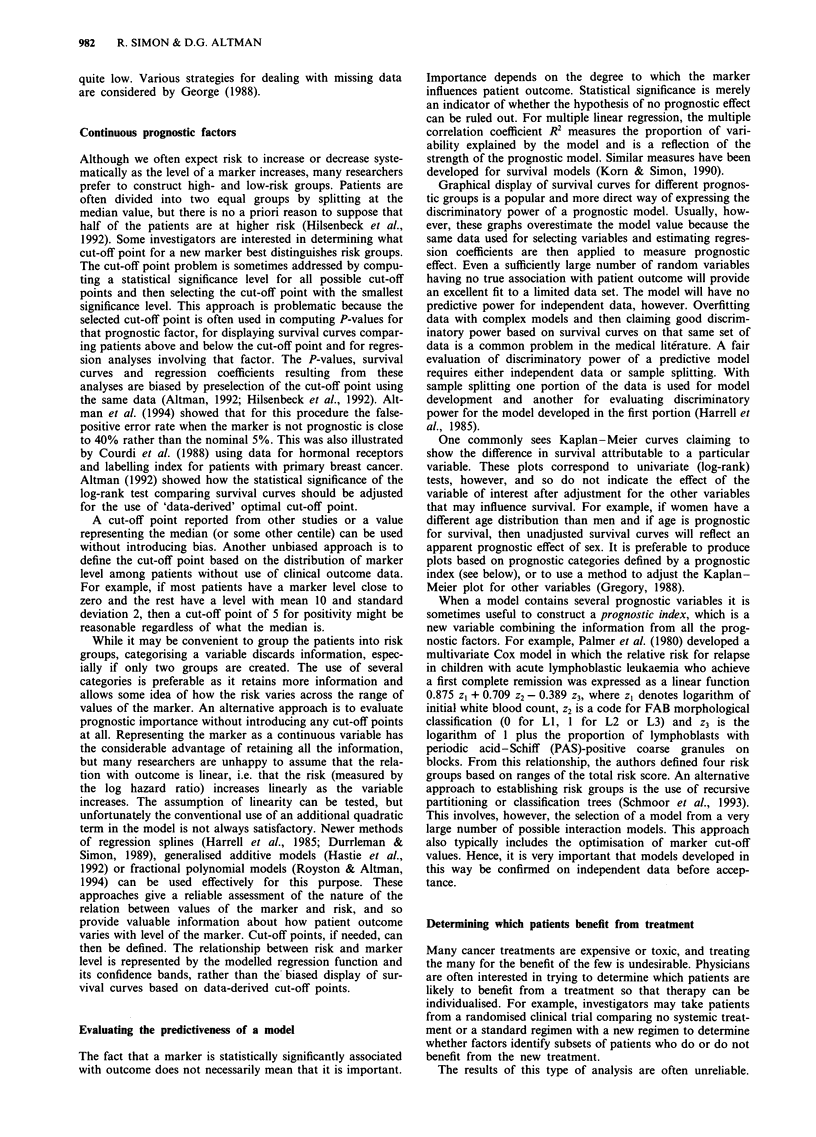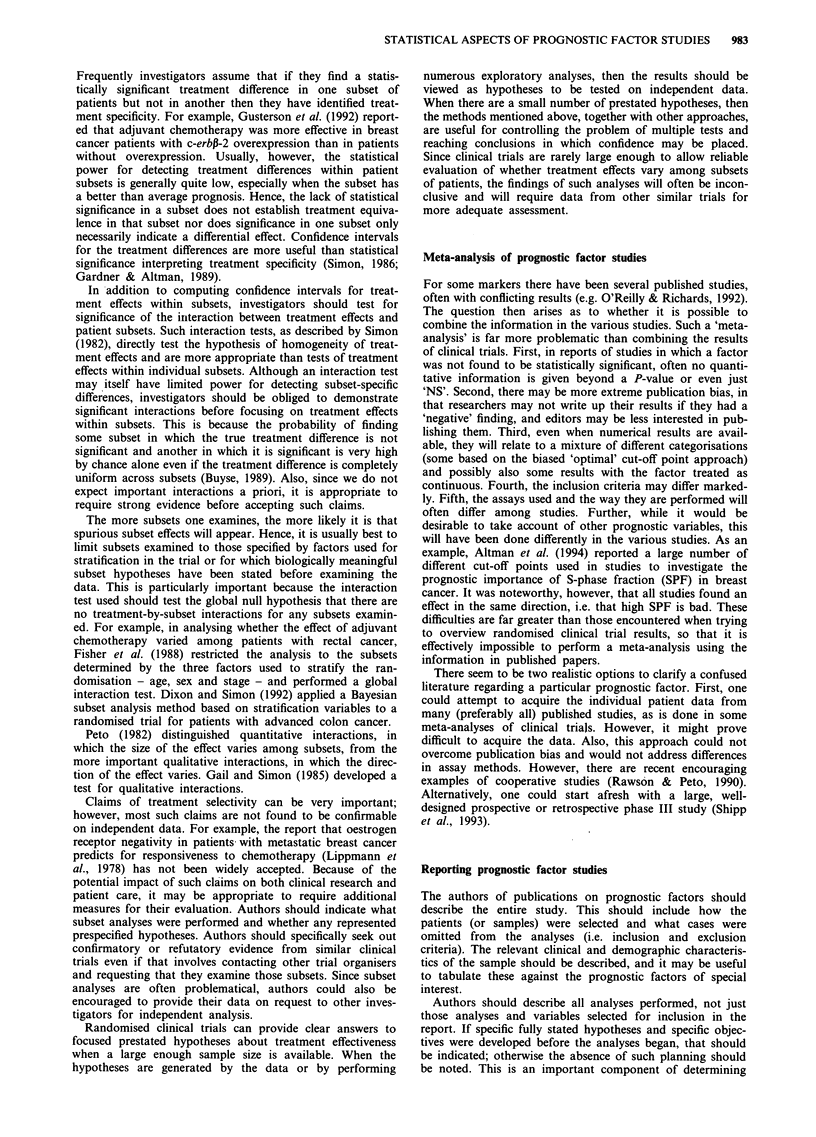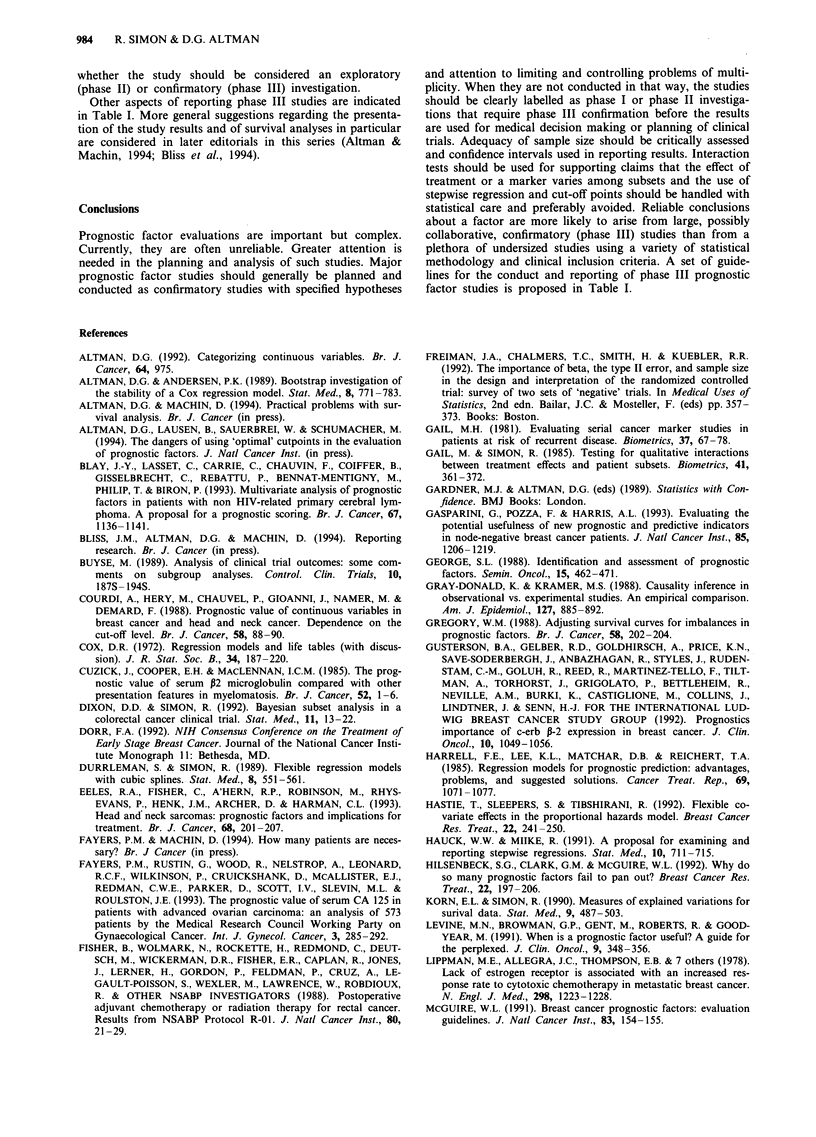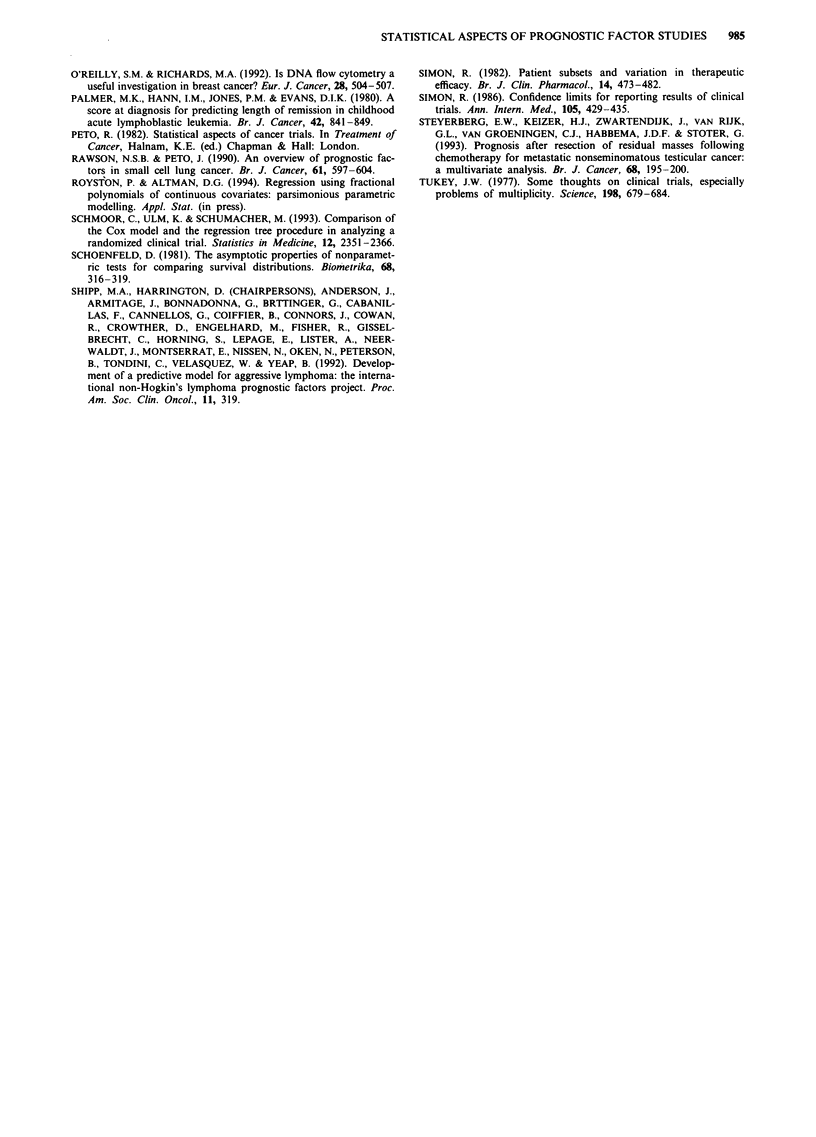# Statistical aspects of prognostic factor studies in oncology.

**DOI:** 10.1038/bjc.1994.192

**Published:** 1994-06

**Authors:** R. Simon, D. G. Altman


Br. J. Cancer (1994), 69, 979-985
© Macmillan Press Ltd., 1994

SPECIAL EDITORIAL SERIES – STATISTICAL ISSUE IN CANCER RESEARCH

### Statistical aspects of prognostic factor studies in oncology

R. Simon[1] & D.G. Altman[2]

[1]*Chief, Biometric Research Branch, National Cancer Institute, 6130 Executive Blvd, Room 739, Rockville, Maryland 20852, USA;* [2]*Head, Medical Statistics Laboratory, Imperial Cancer Research Fund, PO Box 123, Lincoln's Inn Fields, London WC2A 3PX, UK.*

Studies of new prognostic factors form an extensive part of the literature of oncology. Recent examples published in this journal include studies of patients with primary cerebral lymphoma (Blay *et al.*, 1993), head and neck sarcomas (Eeles *et al.*, 1993) and testicular cancer (Steyerberg *et al.*, 1993). In some cases such studies may offer insight into the molecular pathogenesis of the disease. In others they may help in medical decision making; for example, in identifying which patients are at sufficiently high risk of recurrence to warrant a toxic or expensive treatment. Identification of major prognostic determinants can facilitate the design of further clinical trials, aid in inter-trial comparisons and guide the counselling of individual patients. Unfortunately, however, the results of different prognostic factor studies are often inconsistent or contradictory.

Whereas widely accepted methodological principles have evolved to guide the design, conduct, analysis and reporting of clinical trials, no similar guidelines exist for prognostic factor studies. McGuire (1991), Levine *et al.* (1991) and Gasparini *et al.* (1993), discussed some general methodological problems with prognostic factor studies. Here we will consider some of the statistical problems common to prognostic factor studies as a step towards the development of broadly acceptable principles that might guide their conduct and reporting.

### Stating objectives

There are several types of prognostic studies. Most of them can be classified in the following three groups:

1. Early exploratory studies. Such investigations commonly examine issues such as the association of a factor with diagnosis and disease characteristics or the development of reproducible assays.
2. Studies to determine whether prognostic factors provide improved means of identifying patients at particularly high or low risk of disease progression or death.
3. Studies to determine which subsets of patients benefit from a given therapy.

We suggest that studies of type 1 might be called phase I prognostic factor studies. Types 2 and 3 each include what might be called phase II and III factor studies. The phase II studies are exploratory and generate hypotheses from extensive analysis of the data. Phase III studies are large, confirmatory studies of prestated hypotheses, and allow for more precise quantification of the magnitude of the effect.

The report by Gusterson *et al.* (1992) concluded that breast carcinoma tumours which overexpress c-*erbβ*-2 are less responsive to CMF adjuvant therapy than those with a normal amount of gene product. Although this study included specimens collected from 1,506 of the 2,504 patients in a randomised clinical trial, it must be viewed as a phase II study in the sense described here. There is no indication that the focus of the study was on c-*erbβ*-2, although the report is restricted to this oncogene. More importantly, there is no indication that the hypothesis that c-*erbβ*-2 predicted chemoresponsiveness was defined in advance or was suggested by results of a previous study.

The report by Cuzick *et al.* (1985) on the prognostic value of serum $\beta_2$-microglobulin in myelomatosis is an example of a phase III study. The patient population consisted of 476 patients enrolled in the Medical Research Council fourth trial for myelomatosis. The analysis was focused on survival of all entered patients. Serum $\beta_2$-microglobulin was the only new marker evaluated. The results indicated that serum $\beta_2$-microglobulin was the most predictive single factor, but did not address whether predictiveness of the new marker and standard factors was better than that of standard factors alone. Often this will be of considerable importance. The study did not address reproducibility of the $\beta_2$-microglobulin assay and was not based on utilisation of prognostic groups defined in previous studies. The study did not establish the relevance of measuring $\beta_2$-microglobulin outside of clinical trials because it did not test any therapeutic hypothesis involving $\beta_2$-microglobulin.

A principle of good research requires that hypotheses be stated in advance. For example, a hypothesis might be that S-phase fraction greater than 10% is prognostic for disease-free survival over and above the influence of menopausal status, hormonal receptor status and tumour size for node-negative breast cancer patients who have not received adjuvant therapy. Clearly stated hypotheses are, unfortunately, rare in prognostic factor studies. In the terminology defined above, there are very few phase III prognostic factor studies. Numerous analyses with different subsets of factors, different cut-off points for the factors, different subsets of patients and different end points are often performed without specific prestated hypotheses. This approach would not be objectionable if the purpose of the analysis were viewed as screening for interesting hypotheses to be tested in independent data sets; if it were recognised that the results should not be accepted without confirmation. It is unusual, however, that authors interpret the results in that way. Unfortunately, many authors seem unaware of the serious impact of these 'problems of multiplicity'. The probability that some spurious associations will be found is very high. An association giving $P < 0.05$ in a typical exploratory analysis of a set of progostic factors is a much weaker finding than $P < 0.05$ for the primary question in a focused clinical trial (Tukey, 1977), and should not be interpreted in the same way.

Table I lists important components of phase III prognostic factor studies. Several of these components have been described previously by others (e.g. Levine *et al.*, 1991; McGuire, 1991; Fielding *et al.*, 1992; Gasparini *et al.*, 1993). In the sections to follow, we will clarify and discuss some of the statistical issues included in this table.

Our definition of phases describes characteristics of prognostic factors studies but does not directly address the types of studies that should be performed before a marker is accepted for use in clinical practice outside of clinical trials.

Correspondence: R. Simon.
Received 18 February 1994.

This is indicated in Table II in a manner simliar to that suggested by the 1990 National Institutes of Health Consensus Development Conference on the Treatment of Early Stage Breast Cancer (Dorr, 1992). The use of prognostic markers to select excellent-prognosis patients with node-negative breast cancer who do not require adjuvant chemotherapy is currently a controversial area in which practice guidelines would be useful. The marker assay should be demonstrated to be reproducible and widely available with quality control. A clinically important improvement in predictive value of the markers beyond that achievable with recognised prognostic factors should be established. The improved prediction should have easily interpreted therapeutic implications that have been reliably established. And these findings should be substantiated by independently confirmed phase III marker trials.

### Design issues

### *Study design*

Prognostic factors studies can be prospective, in which newly diagnosed patients are entered and followed up for an adequate length of time to allow a comparison of outcome for groups with different baseline values of the factors of interest. Prospective studies are very time-consuming in diseases with relatively good prognosis. More often, prognostic factor studies are retrospective, in which the measurement of interest is made on stored samples (often tumour samples). The advantage of retrospective studies is that information about moderate or long-term follow-up may be available immediately, but the disadvantage is that clinical information may be incomplete. Missing data is a common problem when analysing retrospective data, and the necessary assumption that the values are missing at random is difficult to assess yet is often implausible. Clearly only prospective studies are possible to study a new marker than cannot be measured on stored samples.

It is widely recognised that controlled clinical trials offer the most reliable research evidence. One reason for this is the careful definition of inclusion and exclusion criteria. It has been suggested that the most reliable observational studies are those that try as far as possible to adopt the same careful design standards as are used in clinical trials with the goal of achieving the same answer as if an experimental study had been performed (Gray-Donald & Kramer, 1988). Thus for both prospective and retrospective studies inclusion and exclusion criteria should be carefully defined, so that it is clear to which population of patients the results can reasonably be extrapolated. One problem sometimes encountered with retrospective studies is that tumour specimens are unavailable for many patients and hence the generalisability of conclusions to broad populations is problematic.

Clearly it is also essential that selection of patients should not be related to outcome; this is obviously only an issue for retrospective studies. For retrospective studies there is a risk of bias because stored samples are likely to include a disproportionate number of larger tumours (McGuire, 1991). The characteristics of the sample thus need to be adequately summarised.

**Table I** Guidelines for phase III prognostic factor studies

| | |
|---|---|
| 1. | Intra- and inter-laboratory reproducibility of assays should be documented |
| 2. | Laboratory assays should be performed blinded to clinical data and outcome |
| 3. | A clear inception cohort of patients should be assembled with few (e.g. <15%) patients non-evaluable due to missing material or data. The referral pattern and eligibility criteria should be described so that generalisability of results can be evaluated |
| 4. | Treatment (or absence of treatment) should be standardised or randomised and accounted for in the analysis and interpretation |
| 5. | Hypotheses to be tested should be stated in advance. The hypotheses should include specification of end points, cut-off values for prognostic variables, subsets of patients, treatment, standard prognostic factors or classifications to be used that are relevant to the hypotheses |
| 6. | The number of patients and number of 'events' should be sufficiently large that statistically reliable results are obtainable. Statistical power calculations that incorporate the number of hypotheses to be tested and the appropriate subset of patients (e.g. node-negative) for each hypothesis should be described |
| 7. | Analyses should test whether new assays add predictiveness once outcome is adjusted for the effect of standard prognostic factors |
| 8. | The analyses should be adjusted for the number of hypotheses to be tested |
| 9. | Analyses should be based on prespecified cut-off values for prognostic variables or cut-offs should be avoided |
| 10. | Confidence intervals should be provided to indcate the uncertainty in estimates |
| 11. | Claims of subset-specific treatment effects should be documented by a test of the single global null hypothesis that there is no treatment specificity involving any of the subsetting variables |

**Table II** Requirements of a prognostic marker for acceptance in clinical practice

| | |
|---|---|
| 1. | Determination is reproducible and widely available with quality control |
| 2. | Substantial predictive value beyond recognised prognostic systems is demonstrated |
| 3. | The predictions have therapeutic implications that are readily interpretable by the clinician and of benefit to the patient |
| 4. | Conclusions are based on independently confirmed phase III studies |

### *Sample size*

Most prognostic factor studies are not planned prospectively like clinical trials with specification of hypotheses and sample size determination to assure adequate statistical power. Freiman *et al.* (1992) showed that many clinical trials published as 'negative' (i.e. with a non-significant treatment effect) are really 'uniformative' because of inadequate sample size. The issue of sample size is at least as important for prognostic factor studies because the problems of multiple comparisons in the selection of variables, assessment of their significance and the comparison of models must be considered. Because these issues complicate the determination of appropriate sample size, and because prognostic studies are generally done on available data, sample size planning has received little attention.

Sample size calculations with survival data are complex because several factors, including the length of follow-up and the prevalence of the risk factor in the patient population, need to be considered. Sample size planning for binary end points with survival data can be performed using the method described by Schoenfeld (1981). The required number of events is approximately

$$(z_{1-\alpha} + z_{1-\beta})^2/(\log \mathrm{HR})^2\ w(1-w)$$

where HR denotes the hazard ratio of the prognostic effect, $w$ is the prevalence of the poor risk factor and the constants $z_{1-\alpha}$ and $z_{1-\beta}$ are 1.96 and 1.28 for a two-sided 5% significance level and 90% statistical power. For example, to detect a hazard ratio of 3 if 20% of patients are poor risk requires observing approximately 55 events. The number of patients required approximately equals the number of events required divided by the expected average event rate. For the example given, if the average event rate is only 15% over the course of the study, this requires approximately 370 patients. Because of the number of analyses in many prognostic factor studies, 1% significance is often more appropriate than 5%. In fact, significance should generally only be declared for a phase III study if the computed $P$-value is less than $0.05/c$, where $c$ is the number of comparisons made. The issue of sample size is discussed more completely by Fayers and Machin (1994).

When many variables are to be investigated, further considerations are relevant and will probably override the sort of calculation just discussed. These issues are illustrated by considering the study of Harrell *et al.* (1985), who used regression modelling with stepwise variable selection (discussed in detail later) on a large data set of patients who had undergone cardiac catheterisation. They developed models on a randomly selected 'training' subset of 2,113 patients, of whom 208 had experienced cardiovascular deaths. They developed regression models from a menu of 30 potential prognostic factors in the training set and then examined the degree to which prognostic discrimination deteriorated in an independent test set of data. They found that models developed by stepwise regression on the 30 variables validated very poorly in the 'test' set, also consisting of 2,113 patients and 208 deaths. The models determined on different randomly selected training sets differed considerably. Other authors have found similar instability in Cox regression models applied to survival data (Altman & Andersen, 1989). Harrell *et al.* (1985) concluded that for regression modelling the number of events (e.g. deaths) should be at least ten times the number of potential prognostic variables that could be included in the model. Interactions between prognostic factors and multiple cut-off points (both discussed below) increase the required sample size. The ratio of 10:1 relates number of events to number of prognostic variables studied; it does not represent the relationship between number of patients and number of variables finally selected. Whereas more work is needed in this area, the ratio 10:1 is a reasonable standard at this point for reliable prognostic modelling. Certainly, exploratory phase II studies should be interpreted even more circumspectly when the ratio is lower than 10:1, especially if the sample size is also small.

So far we have considered studies which consider only baseline data, which is the usual case. A rather different study design is to take serial measurements of the same marker at baseline and at several times after treatment. For example, different authors have derived measures of short-term changes in CA125 levels as prognostic markers in advanced ovarian cancer, usually based on rather small data sets. Three such summary measures have recently been compared using pooled data from 11 centres (Fayers *et al.*, 1993). Such studies raise several new design issues on top of those already discussed. Some of these issues were discussed by Gail (1981). For such studies one should prospectively specify the sequential marker change to be tested as an early indicator of recurrence. Sequential marker assays should be performed blinded from clinical information and should remain blinded to clinicians until the final analysis is performed. Very few sequential studies of assays are performed in this manner. As yet we do not think that it is possible to make general recommendations about the design and analysis of such studies.

### Use of stepwise regression

Regression models such as Cox's proportional hazards regression model are often used to study the joint influence of several prognostic factors (Cox, 1972). Sometimes the aim is to examine the effect of a particular 'new' potentially useful variable after allowing for the effect of existing known prognostic factors – in other words, to evaluate not just whether the new factor is prognostic, but whether it adds usefully to existing factors, such as stage. In exploratory (phase II) studies, however, investigators often wish to obtain parsimonious prediction models by excluding the 'less important' factors. In other words, they wish to reduce a set of possible predictors to a small set of 'important' variables. It is customary to use stepwise regression analysis for model selection. With 'forward selection' the first variable included in the model is the one which by itself has the most statistically significant association with patient outcome. The next variable included is that which is most significantly associated with patient outcome after adjustment for the effect of the first variable, etc. Alternatively, with 'backward elimination' all variables are entered into the model, and then the least statistically significant variables are removed one at a time until all the remaining variables are deemed important. The two approaches may lead to different models.

The results of stepwise regression analysis can be difficult to interpret and may be misleading. The main difficulty is that the regression coefficients in the final selected model are biased (they are on average too large) and the significance levels associated with these coefficients are not strictly valid. These effects result from the use of the same data to select the model and to estimate the regression coefficients of the variables appearing in the model. The usual procedures for assessing the statistical significance of a regression coefficient do not account for this selection. It is customary, however, to ignore these issues when interpreting *P*-values from stepwise regression, with the consequent risk of false claims of statistical significance.

Another difficulty is that the order of entry of variables is often interpreted as order of 'importance' of the variables. A variable with two levels, low and high, may effecively distinguish low- from high-risk patients. But if the high level is of low prevalance, the variable will be less significantly associated with outcome, and therefore judged to be less important than another variable whose levels are more balanced but which is less prognostic. Another problem with this definition of importance is that with forward selection the variable selected first will have great influence on what variable is considered second most important. Thus, a variable that is highly correlated with the variable selected first may appear not to be at all important. Further, although it is customary to include variables significant at the 5% level, this is an arbitrary criterion, having no direct relation to clinical importance. Also, the number of variables in the model will tend to increase as the sample size increases (Harrell *et al.*, 1985).

A third problem with stepwise regression is that the variables selected may be highly unstable. That is, minor changes in the data may result in the selection of a different set of predictors. Unfortunately, the labelling of certain variables as important (and, by implication, others as not important) misrepresents the fact that models based on very different sets of factors may predict almost equally well (Hauck & Miike, 1991). If stepwise regression analysis is used, it is desirable for the stability of the model to be evaluated, for example by using new methods such as bootstrap resampling (Altman & Anderson, 1989). With this approach multiple data sets with a structure similar to the original data are simulated, and models are developed on each simulated data set.

In many cases it is desirable to avoid the problems of stepwise regression. If it is important to determine whether a new factor adds prognostic information to that already contained in the more established factors, this should not be addressed by stepwise regression. Rather, a regression model should be fitted containing only the standard factors and then a model fitted containing both the standard factors and the new factor(s). The difference in the fit to the data of the two models provides a measure of statistical significance of whether the new factor(s) contain additional prognostic information. If there are multiple factors, then this approach provides a way of accounting for the number of factors in the calculation of statistical significance because one should first test the single overall hypothesis that none of the factors adds important information. Although such tests of the global null hypothesis are used infrequently, they are important for phase III studies that examine multiple factors. Another difficulty with multiple regression methods, whether or not stepwise procedures are used, is how to deal with missing data. As noted in the section on study design, missing data can be a serious problem, especially in retrospective studies, and the percentage of subjects with full data can be quite low. Various strategies for dealing with missing data are considered by George (1988).

**Continuous prognostic factors**

Although we often expect risk to increase or decrease systematically as the level of a marker increases, many researchers prefer to construct high- and low-risk groups. Patients are often divided into two equal groups by splitting at the median value, but there is no a priori reason to suppose that half of the patients are at higher risk (Hilsenbeck *et al.*, 1992). Some investigators are interested in determining what cut-off point for a new marker best distinguishes risk groups. The cut-off point problem is sometimes addressed by computing a statistical significance level for all possible cut-off points and then selecting the cut-off point with the smallest significance level. This approach is problematic because the selected cut-off point is often used in computing *P*-values for that prognostic factor, for displaying survival curves comparing patients above and below the cut-off point and for regression analyses involving that factor. The *P*-values, survival curves and regression coefficients resulting from these analyses are biased by preselection of the cut-off point using the same data (Altman, 1992; Hilsenbeck *et al.*, 1992). Altman *et al.* (1994) showed that for this procedure the false-positive error rate when the marker is not prognostic is close to 40% rather than the nominal 5%. This was also illustrated by Courdi *et al.* (1988) using data for hormonal receptors and labelling index for patients with primary breast cancer. Altman (1992) showed how the statistical significance of the log-rank test comparing survival curves should be adjusted for the use of 'data-derived' optimal cut-off point.

A cut-off point reported from other studies or a value representing the median (or some other centile) can be used without introducing bias. Another unbiased approach is to define the cut-off point based on the distribution of marker level among patients without use of clinical outcome data. For example, if most patients have a marker level close to zero and the rest have a level with mean 10 and standard deviation 2, then a cut-off point of 5 for positivity might be reasonable regardless of what the median is.

While it may be convenient to group the patients into risk groups, categorising a variable discards information, especially if only two groups are created. The use of several categories is preferable as it retains more information and allows some idea of how the risk varies across the range of values of the marker. An alternative approach is to evaluate prognostic importance without introducing any cut-off points at all. Representing the marker as a continuous variable has the considerable advantage of retaining all the information, but many researchers are unhappy to assume that the relation with outcome is linear, i.e. that the risk (measured by the log hazard ratio) increases linearly as the variable increases. The assumption of linearity can be tested, but unfortunately the conventional use of an additional quadratic term in the model is not always satisfactory. Newer methods of regression splines (Harrell *et al.*, 1985; Durrleman & Simon, 1989), generalised additive models (Hastie *et al.*, 1992) or fractional polynomial models (Royston & Altman, 1994) can be used effectively for this purpose. These approaches give a reliable assessment of the nature of the relation between values of the marker and risk, and so provide valuable information about how patient outcome varies with level of the marker. Cut-off points, if needed, can then be defined. The relationship between risk and marker level is represented by the modelled regression function and its confidence bands, rather than the biased display of survival curves based on data-derived cut-off points.

**Evaluating the predictiveness of a model**

The fact that a marker is statistically significantly associated with outcome does not necessarily mean that it is important. Importance depends on the degree to which the marker influences patient outcome. Statistical significance is merely an indicator of whether the hypothesis of no prognostic effect can be ruled out. For multiple linear regression, the multiple correlation coefficient $R^2$ measures the proportion of variability explained by the model and is a reflection of the strength of the prognostic model. Similar measures have been developed for survival models (Korn & Simon, 1990).

Graphical display of survival curves for different prognostic groups is a popular and more direct way of expressing the discriminatory power of a prognostic model. Usually, however, these graphs overestimate the model value because the same data used for selecting variables and estimating regression coefficients are then applied to measure prognostic effect. Even a sufficiently large number of random variables having no true association with patient outcome will provide an excellent fit to a limited data set. The model will have no predictive power for independent data, however. Overfitting data with complex models and then claiming good discriminatory power based on survival curves on that same set of data is a common problem in the medical literature. A fair evaluation of discriminatory power of a predictive model requires either independent data or sample splitting. With sample splitting one portion of the data is used for model development and another for evaluating discriminatory power for the model developed in the first portion (Harrell *et al.*, 1985).

One commonly sees Kaplan–Meier curves claiming to show the difference in survival attributable to a particular variable. These plots correspond to univariate (log-rank) tests, however, and so do not indicate the effect of the variable of interest after adjustment for the other variables that may influence survival. For example, if women have a different age distribution than men and if age is prognostic for survival, then unadjusted survival curves will reflect an apparent prognostic effect of sex. It is preferable to produce plots based on prognostic categories defined by a prognostic index (see below), or to use a method to adjust the Kaplan–Meier plot for other variables (Gregory, 1988).

When a model contains several prognostic variables it is sometimes useful to construct a *prognostic index*, which is a new variable combining the information from all the prognostic factors. For example, Palmer *et al.* (1980) developed a multivariate Cox model in which the relative risk for relapse in children with acute lymphoblastic leukaemia who achieve a first complete remission was expressed as a linear function $0.875\ z_1 + 0.709\ z_2 - 0.389\ z_3$, where $z_1$ denotes logarithm of initial white blood count, $z_2$ is a code for FAB morphological classification (0 for L1, 1 for L2 or L3) and $z_3$ is the logarithm of 1 plus the proportion of lymphoblasts with periodic acid–Schiff (PAS)-positive coarse granules on blocks. From this relationship, the authors defined four risk groups based on ranges of the total risk score. An alternative approach to establishing risk groups is the use of recursive partitioning or classification trees (Schmoor *et al.*, 1993). This involves, however, the selection of a model from a very large number of possible interaction models. This approach also typically includes the optimisation of marker cut-off values. Hence, it is very important that models developed in this way be confirmed on independent data before acceptance.

**Determining which patients benefit from treatment**

Many cancer treatments are expensive or toxic, and treating the many for the benefit of the few is undesirable. Physicians are often interested in trying to determine which patients are likely to benefit from a treatment so that therapy can be individualised. For example, investigators may take patients from a randomised clinical trial comparing no systemic treatment or a standard regimen with a new regimen to determine whether factors identify subsets of patients who do or do not benefit from the new treatment.

The results of this type of analysis are often unreliable.

Frequently investigators assume that if they find a statistically significant treatment difference in one subset of patients but not in another then they have identified treatment specificity. For example, Gusterson *et al.* (1992) reported that adjuvant chemotherapy was more effective in breast cancer patients with c-*erbβ*-2 overexpression than in patients without overexpression. Usually, however, the statistical power for detecting treatment differences within patient subsets is generally quite low, especially when the subset has a better than average prognosis. Hence, the lack of statistical significance in a subset does not establish treatment equivalence in that subset nor does significance in one subset only necessarily indicate a differential effect. Confidence intervals for the treatment differences are more useful than statistical significance interpreting treatment specificity (Simon, 1986; Gardner & Altman, 1989).

In addition to computing confidence intervals for treatment effects within subsets, investigators should test for significance of the interaction between treatment effects and patient subsets. Such interaction tests, as described by Simon (1982), directly test the hypothesis of homogeneity of treatment effects and are more appropriate than tests of treatment effects within individual subsets. Although an interaction test may itself have limited power for detecting subset-specific differences, investigators should be obliged to demonstrate significant interactions before focusing on treatment effects within subsets. This is because the probability of finding some subset in which the true treatment difference is not significant and another in which it is significant is very high by chance alone even if the treatment difference is completely uniform across subsets (Buyse, 1989). Also, since we do not expect important interactions a priori, it is appropriate to require strong evidence before accepting such claims.

The more subsets one examines, the more likely it is that spurious subset effects will appear. Hence, it is usually best to limit subsets examined to those specified by factors used for stratification in the trial or for which biologically meaningful subset hypotheses have been stated before examining the data. This is particularly important because the interaction test used should test the global null hypothesis that there are no treatment-by-subset interactions for any subsets examined. For example, in analysing whether the effect of adjuvant chemotherapy varied among patients with rectal cancer, Fisher *et al.* (1988) restricted the analysis to the subsets determined by the three factors used to stratify the randomisation – age, sex and stage – and performed a global interaction test. Dixon and Simon (1992) applied a Bayesian subset analysis method based on stratification variables to a randomised trial for patients with advanced colon cancer.

Peto (1982) distinguished quantitative interactions, in which the size of the effect varies among subsets, from the more important qualitative interactions, in which the direction of the effect varies. Gail and Simon (1985) developed a test for qualitative interactions.

Claims of treatment selectivity can be very important; however, most such claims are not found to be confirmable on independent data. For example, the report that oestrogen receptor negativity in patients with metastatic breast cancer predicts for responsiveness to chemotherapy (Lippmann *et al.*, 1978) has not been widely accepted. Because of the potential impact of such claims on both clinical research and patient care, it may be appropriate to require additional measures for their evaluation. Authors should indicate what subset analyses were performed and whether any represented prespecified hypotheses. Authors should specifically seek out confirmatory or refutatory evidence from similar clinical trials even if that involves contacting other trial organisers and requesting that they examine those subsets. Since subset analyses are often problematical, authors could also be encouraged to provide their data on request to other investigators for independent analysis.

Randomised clinical trials can provide clear answers to focused prestated hypotheses about treatment effectiveness when a large enough sample size is available. When the hypotheses are generated by the data or by performing numerous exploratory analyses, then the results should be viewed as hypotheses to be tested on independent data. When there are a small number of prestated hypotheses, then the methods mentioned above, together with other approaches, are useful for controlling the problem of multiple tests and reaching conclusions in which confidence may be placed. Since clinical trials are rarely large enough to allow reliable evaluation of whether treatment effects vary among subsets of patients, the findings of such analyses will often be inconclusive and will require data from other similar trials for more adequate assessment.

### Meta-analysis of prognostic factor studies

For some markers there have been several published studies, often with conflicting results (e.g. O'Reilly & Richards, 1992). The question then arises as to whether it is possible to combine the information in the various studies. Such a 'meta-analysis' is far more problematic than combining the results of clinical trials. First, in reports of studies in which a factor was not found to be statistically significant, often no quantitative information is given beyond a *P*-value or even just 'NS'. Second, there may be more extreme publication bias, in that researchers may not write up their results if they had a 'negative' finding, and editors may be less interested in publishing them. Third, even when numerical results are available, they will relate to a mixture of different categorisations (some based on the biased 'optimal' cut-off point approach) and possibly also some results with the factor treated as continuous. Fourth, the inclusion criteria may differ markedly. Fifth, the assays used and the way they are performed will often differ among studies. Further, while it would be desirable to take account of other prognostic variables, this will have been done differently in the various studies. As an example, Altman *et al.* (1994) reported a large number of different cut-off points used in studies to investigate the prognostic importance of S-phase fraction (SPF) in breast cancer. It was noteworthy, however, that all studies found an effect in the same direction, i.e. that high SPF is bad. These difficulties are far greater than those encountered when trying to overview randomised clinical trial results, so that it is effectively impossible to perform a meta-analysis using the information in published papers.

There seem to be two realistic options to clarify a confused literature regarding a particular prognostic factor. First, one could attempt to acquire the individual patient data from many (preferably all) published studies, as is done in some meta-analyses of clinical trials. However, it might prove difficult to acquire the data. Also, this approach could not overcome publication bias and would not address differences in assay methods. However, there are recent encouraging examples of cooperative studies (Rawson & Peto, 1990). Alternatively, one could start afresh with a large, well-designed prospective or retrospective phase III study (Shipp *et al.*, 1993).

### Reporting prognostic factor studies

The authors of publications on prognostic factors should describe the entire study. This should include how the patients (or samples) were selected and what cases were omitted from the analyses (i.e. inclusion and exclusion criteria). The relevant clinical and demographic characteristics of the sample should be described, and it may be useful to tabulate these against the prognostic factors of special interest.

Authors should describe all analyses performed, not just those analyses and variables selected for inclusion in the report. If specific fully stated hypotheses and specific objectives were developed before the analyses began, that should be indicated; otherwise the absence of such planning should be noted. This is an important component of determining whether the study should be considered an exploratory (phase II) or confirmatory (phase III) investigation.

Other aspects of reporting phase III studies are indicated in Table I. More general suggestions regarding the presentation of the study results and of survival analyses in particular are considered in later editorials in this series (Altman & Machin, 1994; Bliss *et al.*, 1994).

### Conclusions

Prognostic factor evaluations are important but complex. Currently, they are often unreliable. Greater attention is needed in the planning and analysis of such studies. Major prognostic factor studies should generally be planned and conducted as confirmatory studies with specified hypotheses and attention to limiting and controlling problems of multiplicity. When they are not conducted in that way, the studies should be clearly labelled as phase I or phase II investigations that require phase III confirmation before the results are used for medical decision making or planning of clinical trials. Adequacy of sample size should be critically assessed and confidence intervals used in reporting results. Interaction tests should be used for supporting claims that the effect of treatment or a marker varies among subsets and the use of stepwise regression and cut-off points should be handled with statistical care and preferably avoided. Reliable conclusions about a factor are more likely to arise from large, possibly collaborative, confirmatory (phase III) studies than from a plethora of undersized studies using a variety of statistical methodology and clinical inclusion criteria. A set of guidelines for the conduct and reporting of phase III prognostic factor studies is proposed in Table I.

### References

ALTMAN, D.G. (1992). Categorizing continuous variables. *Br. J. Cancer*, **64**, 975.

ALTMAN, D.G. & ANDERSEN, P.K. (1989). Bootstrap investigation of the stability of a Cox regression model. *Stat. Med.*, **8**, 771–783.

ALTMAN, D.G. & MACHIN, D. (1994). Practical problems with survival analysis. *Br. J. Cancer* (in press).

ALTMAN, D.G., LAUSEN, B., SAUERBREI, W. & SCHUMACHER, M. (1994). The dangers of using 'optimal' cutpoints in the evaluation of prognostic factors. *J. Natl Cancer Inst.* (in press).

BLAY, J.-Y., LASSET, C., CARRIE, C., CHAUVIN, F., COIFFER, B., GISSELBRECHT, C., REBATTU, P., BENNAT-MENTIGNY, M., PHILIP, T. & BIRON, P. (1993). Multivariate analysis of prognostic factors in patients with non HIV-related primary cerebral lymphoma. A proposal for a prognostic scoring. *Br. J. Cancer*, **67**, 1136–1141.

BLISS, J.M., ALTMAN, D.G. & MACHIN, D. (1994). Reporting research. *Br. J. Cancer* (in press).

BUYSE, M. (1989). Analysis of clinical trial outcomes: some comments on subgroup analyses. *Control. Clin. Trials*, **10**, 187S–194S.

COURDI, A., HERY, M., CHAUVEL, P., GIOANNI, J., NAMER, M. & DEMARD, F. (1988). Prognostic value of continuous variables in breast cancer and head and neck cancer. Dependence on the cut-off level. *Br. J. Cancer*, **58**, 88–90.

COX, D.R. (1972). Regression models and life tables (with discussion). *J. R. Stat. Soc. B.*, **34**, 187–220.

CUZICK, J., COOPER, E.H. & MACLENNAN, I.C.M. (1985). The prognostic value of serum β2 microglobulin compared with other presentation features in myelomatosis. *Br. J. Cancer*, **52**, 1–6.

DIXON, D.D. & SIMON, R. (1992). Bayesian subset analysis in a colorectal cancer clinical trial. *Stat. Med.*, **11**, 13–22.

DORR, F.A. (1992). *NIH Consensus Conference on the Treatment of Early Stage Breast Cancer*. Journal of the National Cancer Institute Monograph 11: Bethesda, MD.

DURRLEMAN, S. & SIMON, R. (1989). Flexible regression models with cubic splines. *Stat. Med.*, **8**, 551–561.

EELES, R.A., FISHER, C., A'HERN, R.P., ROBINSON, M., RHYS-EVANS, P., HENK, J.M., ARCHER, D. & HARMAN, C.L. (1993). Head and neck sarcomas: prognostic factors and implications for treatment. *Br. J. Cancer*, **68**, 201–207.

FAYERS, P.M. & MACHIN, D. (1994). How many patients are necessary? *Br J Cancer* (in press).

FAYERS, P.M., RUSTIN, G., WOOD, R., NELSTROP, A., LEONARD, R.C.F., WILKINSON, P., CRUICKSHANK, D., MCALLISTER, E.J., REDMAN, C.W.E., PARKER, D., SCOTT, I.V., SLEVIN, M.L. & ROULSTON, J.E. (1993). The prognostic value of serum CA 125 in patients with advanced ovarian carcinoma: an analysis of 573 patients by the Medical Research Council Working Party on Gynaecological Cancer. *Int. J. Gynecol. Cancer*, **3**, 285–292.

FISHER, B., WOLMARK, N., ROCKETTE, H., REDMOND, C., DEUTSCH, M., WICKERMAN, D.R., FISHER, E.R., CAPLAN, R., JONES, J., LERNER, H., GORDON, P., FELDMAN, P., CRUZ, A., LEGAULT-POISSON, S., WEXLER, M., LAWRENCE, W., ROBDIOUX, R. & OTHER NSABP INVESTIGATORS (1988). Postoperative adjuvant chemotherapy or radiation therapy for rectal cancer. Results from NSABP Protocol R-01. *J. Natl Cancer Inst.*, **80**, 21–29.

FREIMAN, J.A., CHALMERS, T.C., SMITH, H. & KUEBLER, R.R. (1992). The importance of beta, the type II error, and sample size in the design and interpretation of the randomized controlled trial: survey of two sets of 'negative' trials. In *Medical Uses of Statistics*, 2nd edn. Bailar, J.C. & Mosteller, F. (eds) pp. 357–373. Books: Boston.

GAIL, M.H. (1981). Evaluating serial cancer marker studies in patients at risk of recurrent disease. *Biometrics*, **37**, 67–78.

GAIL, M. & SIMON, R. (1985). Testing for qualitative interactions between treatment effects and patient subsets. *Biometrics*, **41**, 361–372.

GARDNER, M.J. & ALTMAN, D.G. (eds) (1989). *Statistics with Confidence*. BMJ Books: London.

GASPARINI, G., POZZA, F. & HARRIS, A.L. (1993). Evaluating the potential usefulness of new prognostic and predictive indicators in node-negative breast cancer patients. *J. Natl Cancer Inst.*, **85**, 1206–1219.

GEORGE, S.L. (1988). Identification and assessment of prognostic factors. *Semin. Oncol.*, **15**, 462–471.

GRAY-DONALD, K. & KRAMER, M.S. (1988). Causality inference in observational vs. experimental studies. An empirical comparison. *Am. J. Epidemiol.*, **127**, 885–892.

GREGORY, W.M. (1988). Adjusting survival curves for imbalances in prognostic factors. *Br. J. Cancer*, **58**, 202–204.

GUSTERSON, B.A., GELBER, R.D., GOLDHIRSCH, A., PRICE, K.N., SAVE-SODERBERGH, J., ANBAZHAGAN, R., STYLES, J., RUDENSTAM, C.-M., GOLUH, R., REED, R., MARTINEZ-TELLO, F., TILTMAN, A., TORHORST, J., GRIGOLATO, P., BETTLEHEIM, R., NEVILLE, A.M., BURKI, K., CASTIGLIONE, M., COLLINS, J., LINDTNER, J. & SENN, H.-J. FOR THE INTERNATIONAL LUDWIG BREAST CANCER STUDY GROUP (1992). Prognostics importance of c-erb β-2 expression in breast cancer. *J. Clin. Oncol.*, **10**, 1049–1056.

HARRELL, F.E., LEE, K.L., MATCHAR, D.B. & REICHERT, T.A. (1985). Regression models for prognostic prediction: advantages, problems, and suggested solutions. *Cancer Treat. Rep.*, **69**, 1071–1077.

HASTIE, T., SLEEPERS, S. & TIBSHIRANI, R. (1992). Flexible covariate effects in the proportional hazards model. *Breast Cancer Res. Treat.*, **22**, 241–250.

HAUCK, W.W. & MIIKE, R. (1991). A proposal for examining and reporting stepwise regressions. *Stat. Med.*, **10**, 711–715.

HILSENBECK, S.G., CLARK, G.M. & MCGUIRE, W.L. (1992). Why do so many prognostic factors fail to pan out? *Breast Cancer Res. Treat.*, **22**, 197–206.

KORN, E.L. & SIMON, R. (1990). Measures of explained variations for surival data. *Stat. Med.*, **9**, 487–503.

LEVINE, M.N., BROWMAN, G.P., GENT, M., ROBERTS, R. & GOODYEAR, M. (1991). When is a prognostic factor useful? A guide for the perplexed. *J. Clin. Oncol.*, **9**, 348–356.

LIPPMAN, M.E., ALLEGRA, J.C., THOMPSON, E.B. & 7 others (1978). Lack of estrogen receptor is associated with an increased response rate to cytotoxic chemotherapy in metastatic breast cancer. *N. Engl. J. Med.*, **298**, 1223–1228.

MCGUIRE, W.L. (1991). Breast cancer prognostic factors: evaluation guidelines. *J. Natl Cancer Inst.*, **83**, 154–155.

O'REILLY, S.M. & RICHARDS, M.A. (1992). Is DNA flow cytometry a useful investigation in breast cancer? *Eur. J. Cancer*, **28**, 504–507.

PALMER, M.K., HANN, I.M., JONES, P.M. & EVANS, D.I.K. (1980). A score at diagnosis for predicting length of remission in childhood acute lymphoblastic leukemia. *Br. J. Cancer*, **42**, 841–849.

PETO, R. (1982). Statistical aspects of cancer trials. In *Treatment of Cancer*, Halnam, K.E. (ed.) Chapman & Hall: London.

RAWSON, N.S.B. & PETO, J. (1990). An overview of prognostic factors in small cell lung cancer. *Br. J. Cancer*, **61**, 597–604.

ROYSTON, P. & ALTMAN, D.G. (1994). Regression using fractional polynomials of continuous covariates: parsimonious parametric modelling. *Appl. Stat.* (in press).

SCHMOOR, C., ULM, K. & SCHUMACHER, M. (1993). Comparison of the Cox model and the regression tree procedure in analyzing a randomized clinical trial. *Statistics in Medicine*, **12**, 2351–2366.

SCHOENFELD, D. (1981). The asymptotic properties of nonparametric tests for comparing survival distributions. *Biometrika*, **68**, 316–319.

SHIPP, M.A., HARRINGTON, D. (CHAIRPERSONS), ANDERSON, J., ARMITAGE, J., BONNADONNA, G., BRTTINGER, G., CABANILLAS, F., CANNELLOS, G., COIFFIER, B., CONNORS, J., COWAN, R., CROWTHER, D., ENGELHARD, M., FISHER, R., GISSELBRECHT, C., HORNING, S., LEPAGE, E., LISTER, A., NEERWALDT, J., MONTSERRAT, E., NISSEN, N., OKEN, N., PETERSON, B., TONDINI, C., VELASQUEZ, W. & YEAP, B. (1992). Development of a predictive model for aggressive lymphoma: the international non-Hogkin's lymphoma prognostic factors project. *Proc. Am. Soc. Clin. Oncol.*, **11**, 319.

SIMON, R. (1982). Patient subsets and variation in therapeutic efficacy. *Br. J. Clin. Pharmacol.*, **14**, 473–482.

SIMON, R. (1986). Confidence limits for reporting results of clinical trials. *Ann. Intern. Med.*, **105**, 429–435.

STEYERBERG, E.W., KEIZER, H.J., ZWARTENDIJK, J., VAN RIJK, G.L., VAN GROENINGEN, C.J., HABBEMA, J.D.F. & STOTER, G. (1993). Prognosis after resection of residual masses following chemotherapy for metastatic nonseminomatous testicular cancer: a multivariate analysis. *Br. J. Cancer*, **68**, 195–200.

TUKEY, J.W. (1977). Some thoughts on clinical trials, especially problems of multiplicity. *Science*, **198**, 679–684.